# A Two‐Step PCR Workflow Reveals Predominance of *Flavobacterium covae* in Ornamental Fish With Clinical Signs of Columnaris Disease

**DOI:** 10.1111/jfd.70228

**Published:** 2026-06-26

**Authors:** Pedro Henrique Magalhães Cardoso, Emy Yano, Simone de Carvalho Balian, André Pegoraro Poor, Kawany Miyazaki Araujo, Marianna Vaz Rodrigues, Andrea Micke Moreno

**Affiliations:** ^1^ Department of Preventive Veterinary Medicine and Animal Health, School of Veterinary Medicine and Animal Science University of São Paulo São Paulo Brazil; ^2^ Institute of Biotechnology São Paulo State University “Júlio de Mesquita Filho” Botucatu Brazil

**Keywords:** columnaris disease, *Flavobacterium covae*, *Flavobacterium davisii*, multiplex PCR, ornamental fish

## Abstract

Columnaris is an important bacterial disease of freshwater fish and is increasingly recognised as being caused by a species complex historically referred to as *Flavobacterium columnare* . A two‐step molecular workflow was applied to ornamental fish with clinical signs of columnaris disease from a multispecies commercial distributor operating a shared recirculating system in São Paulo, Brazil. Skin and fin lesion scrapings were Gram stained, and lesion‐derived tissue DNA was screened by ISR‐PCR for the columnaris disease complex. ISR‐PCR yielded the expected amplicon in 61/123 samples (49.6%) from 28 ornamental fish species. All ISR‐PCR‐positive samples were then analysed by multiplex PCR for species assignment. Group‐specific amplicons were obtained from 29/61 samples (47.5%): *Flavobacterium covae* in 27/61 (44.3%) and *F. davisii* in 2/61 (3.3%); no GG1 or GG4 amplicons were detected. The remaining 32/61 samples (52.5%) were not typeable by multiplex PCR. These findings provide baseline species‐level data and support combined molecular surveillance in multispecies ornamental fish systems.

## 1 Introduction

In ornamental fish distribution facilities, inadequate biosecurity and shared recirculating water systems can facilitate pathogen dissemination, especially when fish from different sources are housed in interconnected tanks (Cardoso et al. 2019). Accurate pathogen identification is therefore essential for disease management, surveillance and targeted biosecurity measures (Dominguez et al. 2023). Columnaris is an important bacterial disease of freshwater fish, associated with external skin, fin and gill lesions and the presence of long, slender Gram‐negative rods in affected tissues (Declercq et al. 2013). Microscopy supports a presumptive diagnosis, but PCR‐based assays can support confirmation of the columnaris disease complex and subsequent species‐level investigation (Welker et al. 2005). Genome‐based studies have shown that the historical taxon *Flavobacterium columnare* comprises a species complex including *F. columnare* , *F. covae*, *F. davisii* and *F. oreochromis*, which has implications for diagnostic interpretation (LaFrentz et al. 2022; Han et al. 2023). ISR‐PCR can detect members of the complex without species‐level assignment (Welker et al. 2005), whereas multiplex PCR can discriminate genetic groups within it (LaFrentz et al. 2019). This study used a two‐step molecular workflow, combining ISR‐PCR screening and multiplex PCR genotyping, to generate baseline data on the occurrence and species distribution of columnaris‐associated *Flavobacterium* in diseased ornamental fish from a shared recirculating commercial system.

## 2 Materials and Methods

### 2.1 Animals

All procedures were approved by the Institutional Committee for Animal Ethics of the University of São Paulo (CEUA; approval no. 6927180221). A total of 123 ornamental fish representing 28 species were obtained during an outbreak from a wholesale distributor in the Greater São Paulo, Brazil, where fish were maintained in interconnected aquaria within a shared recirculating water system without ozonation or ultraviolet disinfection. Fish showed clinical signs compatible with columnaris disease, including pale or reddened skin and fin lesions (Figure 1A–H), lethargy, anorexia, erratic swimming, and increased opercular movements. Clinically affected fish were submitted for diagnostic assessment to the Department of Preventive Veterinary Medicine and Animal Health, School of Veterinary Medicine and Animal Science, University of São Paulo, in May 2021. Before sedation and external disinfection, skin and fin scrapings from gross lesions were Gram stained. Long, slender Gram‐negative rods were observed (Figure 1I), consistent with *Flavobacterium*‐like morphology. Bacterial isolation was not performed; therefore, microscopy was used as a supportive screening step before molecular analyses. Fish were sedated with eugenol at 100 mg L^−1^ for 2–6 min and euthanised by spinal cord transection (Roubach et al. 2005; Noga 2010). After euthanasia, fish were rinsed with 70% alcohol to reduce potential surface contamination, and lesioned skin tissue was collected for molecular analysis. Samples were stored in microtubes at −20°C until molecular processing.

**FIGURE 1:**
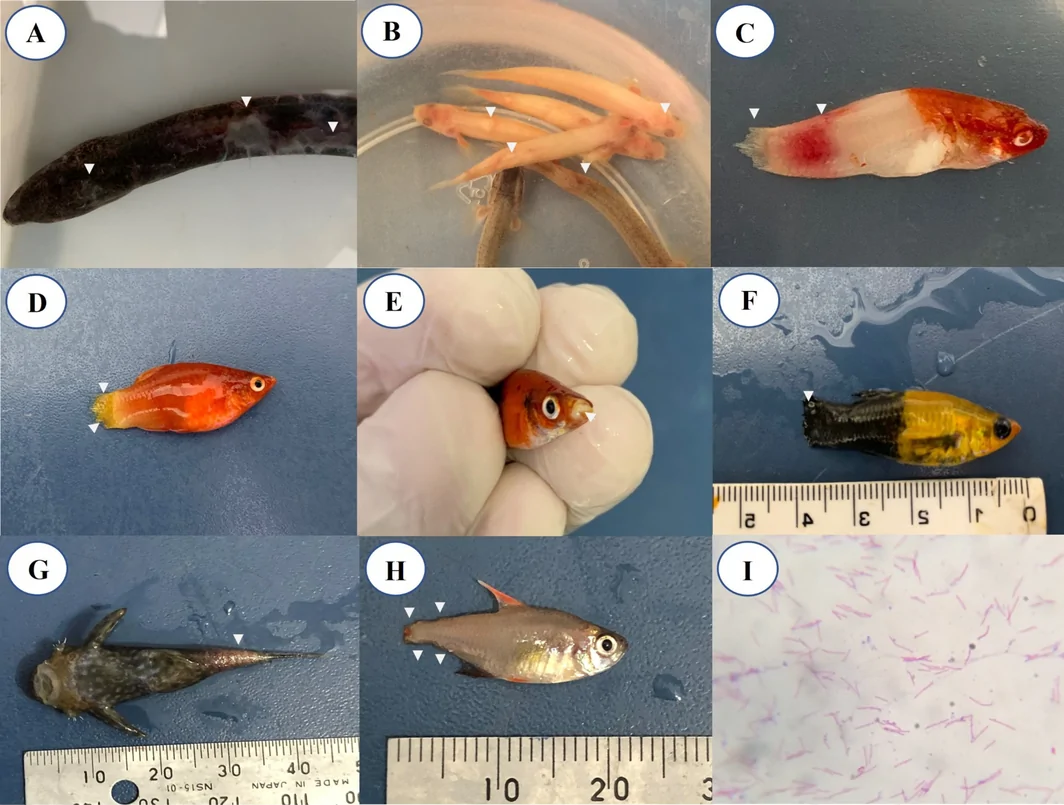
Clinical signs of opaque skin lesions in *Synbranchus marmoratus* (marbled swamp eel) (A); *Polypterus senegalus* (grey bichir) (B); reddish lesion on the caudal peduncle of *Xiphophorus hellerii* (green swordtail) (C); lesion on the caudal fin of *Xiphophorus maculatus* (southern platyfish) (D); opaque lesion in the oral cavity of *X. maculatus* (E); lesion on the caudal peduncle of *Poecilia sphenops* (molly) (F); reddish lesion on the caudal peduncle of *Ancistrus cirrhosus* (G); opaque lesions at the caudal end in *Hyphessobrycon eques* (serpae tetra) (H); and Gram‐stained scraping smear showing long Gram‐negative rods (I).

### 2.2 DNA Extraction

DNA was extracted from 1 mL of macerated tissue following Boom et al. (1990), and the extracts were stored at −20°C until PCR analysis.

### 2.3 PCR Screening of Clinical Samples

Tissue samples from clinically affected fish were labelled 1–123 and homogenised in saline. ISR‐PCR screening was performed with primers FCISRFL and FCISRR1 targeting the 16S–23S rDNA intergenic spacer region to detect the columnaris disease complex (Welker et al. 2005). Each ISR‐PCR run included a no‐template control and genomic DNA from a laboratory isolate of the columnaris disease complex, FC078/2020, as a positive control. This isolate, maintained in the laboratory microbial culture collection, had previously yielded the expected ISR‐PCR amplicon using the assay described by Welker et al. (2005). PCR runs were considered valid only when the positive control yielded the expected amplicon and the no‐template control showed no amplification. Each 50 μL ISR‐PCR contained 5 μL DNA extract, 1× PCR buffer, 1.5 mM MgCl_2_, 200 μM of each dNTPs, 20 pmol of each primer, 1 U HOT FIREPol DNA polymerase and ultrapure water to volume. Cycling was performed in a C1000 Touch Thermal Cycler (Bio‐Rad) under the following conditions: 94°C for 5 min; 40 cycles of 94°C for 30 s, 55°C for 45 s and 72°C for 60 s; and 72°C for 7 min. Amplicons of the expected size (500–550 bp) were resolved on 1.5% agarose gels stained with BlueGreen, visualised under UV using a Gel Doc XR system, and sized with a 100 bp DNA ladder.

### 2.4 Multiplex PCR for Species Assignment

ISR‐PCR‐positive samples were subjected to multiplex PCR for species assignment within the columnaris disease complex, following LaFrentz et al. (2019) with modifications for tissue‐derived DNA. This assay generates genetic group‐specific amplicons distinguishable by size after agarose gel electrophoresis; primer sequences and expected amplicon sizes are provided in Table S1. Each run included a no‐template control and genomic DNA from the same laboratory isolate used as the ISR‐PCR positive control, FC078/2020, which was subsequently assigned to GG2/*Flavobacterium covae* by multiplex PCR and used as the positive amplification control for the expected 320‐bp amplicon. Multiplex PCR runs were considered valid only when the GG2 positive amplification control yielded the expected 320‐bp amplicon and the no‐template control showed no amplification. Each 50 μL multiplex PCR contained 5 μL DNA template, 1× buffer, 1.5 mM MgCl_2_, 200 μM of each dNTPs, 10 pmol of each primer, 1 U Taq DNA polymerase and ultrapure water to volume. Cycling was performed in a C1000 Touch Thermal Cycler (Bio‐Rad) under the following conditions: 95°C for 15 min; 34 cycles of 95°C for 45 s, 56°C for 40 s and 72°C for 60 s; and 72°C for 10 min. Products were resolved on 1.5% agarose gels in 0.5× TBE, stained with BlueGreen, visualised under UV and sized with a 100 bp DNA ladder.

## 3 Results

### 3.1 ISR‐PCR Screening of the Columnaris Disease Complex Across Ornamental Fish Species

A total of 123 ornamental fish representing 28 species were screened by ISR‐PCR. Overall, 61/123 samples (49.6%) yielded the expected ISR‐PCR amplicon (Table S2). Positivity varied among species, but species‐level proportions should be interpreted cautiously because sample sizes differed markedly among taxa. Detailed species‐level results are provided in Table S2.

### 3.2 Species Assignment by Multiplex PCR Among ISR‐PCR–Positive Samples Within the Columnaris Disease Complex

All ISR‐PCR‐positive samples (*n* = 61) were tested by multiplex PCR genotyping. Group‐specific amplicons were obtained from 29/61 samples (47.5%). Of the 61 ISR‐PCR‐positive samples, 27 (44.3%) yielded the 320‐bp GG2 amplicon consistent with *Flavobacterium covae*, and two (3.3%) yielded the 894‐bp GG3 amplicon consistent with *F. davisii* (Table S3). No GG1 or GG4 amplicons were detected. The remaining 32/61 samples (52.5%) were not typeable by multiplex PCR. Detailed genotyping results are provided in Table S3.

## 4 Discussion

This two‐step molecular workflow detected the columnaris disease complex in ornamental fish from a shared recirculating commercial system and identified *Flavobacterium covae* as the predominant species among typeable samples. In this study, ISR‐PCR was used to screen lesion‐derived DNA for the columnaris disease complex (Welker et al. 2005), whereas multiplex PCR was used for genetic group/species assignment, acknowledging that the latter was originally developed mainly with DNA from pure‐culture isolates (LaFrentz et al. 2019). Thus, the tissue‐based ISR‐PCR used here served as the initial detection step, while multiplex PCR provided species‐level assignments only when group‐specific amplification occurred. Because DNA was extracted directly from lesions, failure of ISR‐PCR‐positive samples to amplify in the multiplex assay could reflect low bacterial DNA concentrations, PCR inhibitors or variable sample quality; these cases were conservatively interpreted as positive for the columnaris disease complex without species‐level assignment. Higher‐resolution confirmation would require bacterial isolation and/or sequencing of discriminatory markers such as *dnaK*.

Although species‐level data on columnaris‐associated *Flavobacterium* in ornamental fish remain limited, the predominance of *F. covae* among typeable samples is consistent with reports from commercial aquaculture systems. In Brazil, Janampa‐Sarmiento et al. (2025) identified *F. davisii*, *F. covae* and *F. oreochromis* among columnaris‐associated isolates from farmed fish and reported no detection of *F. columnare* , suggesting that species composition within the complex may vary according to host and production context. Recent studies have also documented *F. covae* in diagnostic and experimental contexts associated with columnaris disease in commercial aquaculture (LaFrentz et al. 2024; Tuttle et al. 2023; Wise et al. 2025).

Further reports document *F. covae* in a recirculating aquaculture system in Australia (Samsing et al. 2025). In India, outbreak‐associated isolates from rainbow trout were assigned to GG2, which is currently recognised as *F. covae* (Singh et al. 2021). Although direct comparisons among systems are limited by differences in host species, sampling design and diagnostic methods, these reports indicate the recurrent detection of *F. covae* in diverse production settings.

Improved taxonomic resolution within the columnaris disease complex is epidemiologically relevant, especially in multispecies ornamental fish systems. Because the historical taxon *F. columnare* comprises multiple closely related species, reporting detections solely as “ *F. columnare* ” may obscure differences in distribution and epidemiological patterns (LaFrentz et al. 2022). In distribution systems involving the rapid movement of fish from multiple sources, a two‐step workflow combining detection of the complex with species‐ or group‐level assignment can strengthen surveillance, improve diagnostic interpretation and support targeted biosecurity measures, including quarantine and system‐level disinfection (Welker et al. 2005; LaFrentz et al. 2019).

In summary, this study provides baseline molecular evidence of columnaris‐associated *Flavobacterium* in ornamental fish from a multispecies commercial system, with *F. covae* predominating among typeable samples. Interpretation should consider the absence of bacterial isolation, the use of lesion‐derived DNA for molecular analyses, and sampling limited to a single distributor and collection period. Future studies should combine bacterial isolation, sequencing of discriminatory markers such as *dnaK*, histopathology and broader surveillance across facilities and supply‐chain stages to better characterise species distribution, tissue involvement and effective preventive measures.

## 5 Conclusion

In conclusion, columnaris‐associated *Flavobacterium* was frequently detected in ornamental fish with clinical signs of disease maintained in a shared recirculating system, and *F. covae* predominated among typeable samples. The partial typeability observed highlights that screening and genotyping assays address different diagnostic objectives, particularly when applied to lesion‐derived DNA. These findings provide baseline species‐level information for ornamental fish systems and support combined molecular surveillance alongside strengthened biosecurity practices.

## Author Contributions

**Simone de Carvalho Balian:** conceptualization, formal analysis, writing – review and editing. **Andrea Micke Moreno:** conceptualization, methodology, writing – original draft, supervision, investigation, formal analysis. **Emy Yano:** conceptualization, methodology, writing – review and editing, investigation. **Kawany Miyazaki Araujo:** methodology, writing – review and editing, investigation. **Pedro Henrique Magalhães Cardoso:** conceptualization, methodology, formal analysis, writing – original draft, writing – review and editing, supervision, investigation. **Marianna Vaz Rodrigues:** formal analysis, writing – review and editing, investigation. **André Pegoraro Poor:** methodology, writing – review and editing, investigation.

## Funding

This work was supported by Coordenação de Aperfeiçoamento de Pessoal de Nível Superior, 88887.078833/2024‐00, Finance Code 001. Conselho Nacional de Desenvolvimento Científico e Tecnológico, 312684/2022‐3.

## Ethics Statement

All procedures were approved by the Institutional Committee for Animal Ethics of the University of São Paulo (CEUA; approval no. 6927180221).

## Conflicts of Interest

The authors declare no conflicts of interest.

## Supporting information


**Table S1:** Sequences of primers used for the multiplex PCR to *Flavobacterium* species, including amplicon sizes. Information adapted from LaFrentz et al., 2019.
**Table S2:** Species‐level distribution of ISR‐PCR‐positive ornamental fish sampled during a columnaris disease outbreak in a wholesale facility in São Paulo, Brazil.
**Table S3:** Multiplex PCR genotyping results for ISR‐PCR‐positive samples, showing genetic group assignment within the columnaris disease complex.

## Data Availability

The data that support the findings of this study are available from the corresponding author upon reasonable request.
