# Supplementary material for: A Two‐Step PCR Workflow Reveals Predominance of Flavobacterium covae in Ornamental Fish With Clinical Signs of Columnaris Disease
Source: J Fish Dis. 2026 Jun 26;49(11):e70228. doi: 10.1111/jfd.70228 (PMC13629458; doi:10.1111/jfd.70228)
Supplement: Supplementary file 1 [file JFD-49-e70228-s001.docx]

**Supplementary table S1:** Sequences of primers used for the multiplex PCR to *Flavobacterium* species, including amplicon sizes. Information adapted from LaFrentz et al., 2019

| **Primer** | **Targeted *Flavobacterium species*** | **Amplicon size (bp)** | **Sequence (5′ → 3′)** |
| --- | --- | --- | --- |
| GG-fwd | All |  | ACRGGRGATAAAGCAGAASA |
| GG1-rev | *F. columnare* | 415 | GACTTTTGTGTTGAAACGG |
| GG2-ver | *F. covae* | 320 | AAGAAAATAGGGGAGAGG |
| GG3-ver | *F. davisii* | 894 | CAAGTTTCGTTATGATGAGG |
| GG4-rev | *F. oreochromis* | 659 | TCCAAAAAGTCCGMAATC |

**Supplementary Table S2:** Species-level distribution of ISR-PCR-positive ornamental fish sampled during a columnaris disease outbreak in a wholesale facility in São Paulo, Brazil.

| **Fish Species** | **Common name** | **Fish Family** | **Number of analyzed fish** | **Number of PCR positive fish (%)** |
| --- | --- | --- | --- | --- |
| *Arapaima gigas* | Arapaima | Arapaimidae | 10 | 7 (70 %) |
| *Ancistrus cirrhosus* | Ancistrus | Loricariidae | 1 | 1 (100%) |
| *Cichla pinima* | Pinima peacock bass | Cichlidae | 3 | 1 (33 %) |
| *Cyprinus carpio* | Common carp | Cyprinidae | 3 | 2 (67 %) |
| *Eigenmannia virescens* | Glass knifefish | Sternopygidae | 2 | 1 (50 %) |
| *Glossolepis incisa* | Red rainbowfish | Melanotaeniidae | 4 | 1 (25 %) |
| *Gymnocorymbus ternetzi* | Black tetra | Characidae | 4 | 1 (25 %) |
| *Hemichromis bimaculatus* | Jewel cichlid | Cichlidae | 2 | 1 (50 %) |
| *Helostoma temminckii* | Kissing gourami | Helostomatidae | 2 | 2 (100 %) |
| *Hyphessobrycon ecques* | Serpae tetra | Characidae | 9 | 3 (33 %) |
| *Hyphessobrycon herbertaxelrodi* | Black neon tetra | Characidae | 2 | 1 (50%) |
| *Hyphessobrycon wadai* | Wadai tetra | Characidae | 2 | 1 (50 %) |
| *Ictalurus punctatus* | Channel catfish | Ictaluridae | 2 | 1 (50%) |
| *Iriatherina werneri* | Threadfin rainbowfish | Melanotaeniidae | 4 | 1 (25 %) |
| *Lepisosteus oculatus* | Spotted gar | Lepisosteidae | 3 | 1 (25 %) |
| *Melanochromis auratus* | Golden mbuna | Cichlidae | 2 | 1 (50%) |
| *Melanotaenia praecox* | Dwarf neon rainbowfish | Melanotaeniidae | 1 | 1 (100%) |
| *Mikrogeophagus ramirezi* | Ram cichlid | Cichlidae | 5 | 1 (20 %) |
| *Paracheirodon axelrodi* | Cardinal tetra | Characidae | 4 | 2 (50 %) |
| *Poecilia reticulata* | Guppy | Poeciliidae | 8 | 6 (75 %) |
| *Poecilia sphenops* | Molly | Poeciliidae | 1 | 1 (100 %) |
| *Polypterus senegalus* | Senegal bichir | Polypteridae | 4 | 2 (50%) |
| *Pterygoplichthys pardalis* | Amazon sailfin catfish | Loricariidae | 2 | 2 (100 %) |
| *Puntius titeya* | Cherry barb | Cyprinidae | 6 | 4 (67 %) |
| *Synbranchus marmoratus* | Marbled swamp eel | Synbranchidae | 6 | 1 (17 %) |
| *Stigmatogobius sadanundio* | Knight goby | Gobiidae | 4 | 2 (50 %) |
| *Trichogaster microlepis* | Moonlight gourami | Osphronemidae | 6 | 1 (17 %) |
| *Xiphophorus hellerii* | Green swordtail | Poeciliidae | 5 | 2 (40 %) |
| *Xiphophorus maculatus* | Southern platyfish | Poeciliidae | 16 | 10 (62,5 %) |
|  |  | **Total** | 123 | **61** (49,59%) |

**Supplementary Table S3:** Multiplex PCR genotyping results for ISR-PCR-positive samples, showing genetic group assignment within the columnaris disease complex.

| ID Sample | Fish Specie | Conventional PCR for screening by Welker et al., 2005 | Multiplex PCR for species differentiation | Identified species | G1 - 415 bp    *F. columnare* | G2 - 320 bp  *F. covae* | G3 - 894 bp  *F. davisii* | G4 - 659 bp    *F. oreochromis* |
| --- | --- | --- | --- | --- | --- | --- | --- | --- |
| 02 | *Polypterus senegalus* | Positive | Negative | - | - | - | - | - |
| 04 | *Polypterus senegalus* | Positive | Negative | - | - | - | - | - |
| 06 | *Xiphophorus maculatus* | Positive | Positive | *F. covae* | - | + | - | - |
| 07 | *Xiphophorus maculatus* | Positive | Positive | *F. covae* | - | + | - | - |
| 08 | *Xiphophorus maculatus* | Positive | Positive | *F. covae* | - | + | - | - |
| 10 | *Xiphophorus maculatus* | Positive | Negative | - | - | - | - | - |
| 12 | *Gymnocorymbus ternetzi* | Positive | Negative | - | - | - | - | - |
| 11 | *Poecilia reticulata* | Positive | Negative | - | - | - | - | - |
| 16 | *Poecilia reticulata* | Positive | Positive | *F. covae* | - | + | - | - |
| 17 | *Xiphophorus maculatus* | Positive | Negative | - | - | - | - | - |
| 18 | *Xiphophorus maculatus* | Positive | Negative | - | - | - | - | - |
| 20 | *Xiphophorus maculatus* | Positive | Positive | *F. covae* | - | + | - | - |
| 25 | *Xiphophorus hellerii* | Positive | Positive | *F. covae* | - | + | - | - |
| 26 | *Xiphophorus hellerii* | Positive | Positive | *F. covae* | - | + | - | - |
| 27 | *Poecilia sphenops* | Positive | Positive | *F. covae* | - | + | - | - |
| 30 | *Arapaima gigas* | Positive | Negative | - | - | - | - | - |
| 31 | *Arapaima gigas* | Positive | Negative | - | - | - | - | - |
| 32 | *Arapaima gigas* | Positive | Negative | - | - | - | - | - |
| 33 | *Arapaima gigas* | Positive | Negative | - | - | - | - | - |
| 34 | *Arapaima gigas* | Positive | Negative | - | - | - | - | - |
| 38 | *Arapaima gigas* | Positive | Negative | - | - | - | - | - |
| 39 | *Arapaima gigas* | Positive | Negative | - | - | - | - | - |
| 42 | *Poecilia reticulata* | Positive | Negative | - | - | - | - | - |
| 43 | *Poecilia reticulata* | Positive | Negative | - | - | - | - | - |
| 44 | *Poecilia reticulata* | Positive | Positive | *F. covae* | - | + | - | - |
| 45 | *Poecilia reticulata* | Positive | Positive | *F. covae* | - | + | - | - |
| 47 | *Xiphophorus maculatus* | Positive | Positive | *F. covae* | - | + | - | - |
| 48 | *Xiphophorus maculatus* | Positive | Positive | *F. covae* | - | + | - | - |
| 49 | *Xiphophorus maculatus* | Positive | Negative | - | - | - | - | - |
| 52 | *Synbranchus marmoratus* | Positive | Positive | *F. covae* | - | + | - | - |
| 54 | *Hyphessobrycon ecques* | Positive | Negative | - | - | - | - | - |
| 55 | *Iriatherina werneri* | Positive | Negative | - | - | - | - | - |
| 57 | *Hyphessobrycon ecques* | Positive | Positive | *F. covae* | - | + | - | - |
| 58 | *Paracheirodon axelrodi* | Positive | Positive | *F. covae* | - | + | - | - |
| 59 | *Melanotaenia praecox* | Positive | Negative | - | - | - | - | - |
| 63 | *Helostoma temminckii* | Positive | Positive | *F. covae* | - | + | - | - |
| 65 | *Helostoma temminckii* | Positive | Negative | - | - | - | - | - |
| 67 | *Cyprinus carpio* | Positive | Positive | *F. davisii* | - | - | + | - |
| 68 | *Cyprinus carpio* | Positive | Negative | - | - | - | - | - |
| 69 | *Ictalurus punctatus* | Positive | Positive | *F. covae* | - | + | - | - |
| 70 | *Mikrogeophagus ramirezi* | Positive | Negative | - | - | - | - | - |
| 73 | *Pterygoplichthys pardalis* | Positive | Negative | - | - | - | - | - |
| 74 | *Pterygoplichthys pardalis* | Positive | Negative | - | - | - | - | - |
| 78 | *Glossolepis incisa* | Positive | Positive | *F. covae* | - | + | - | - |
| 79 | *Hyphessobrycon wadai* | Positive | Positive | *F. covae* | - | + | - | - |
| 81 | *Paracheirodon axelrodi* | Positive | Negative | - | - | - | - | - |
| 83 | *Hemichromis bimaculatus* | Positive | Negative | - | - | - | - | - |
| 84 | *Puntius titeya* | Positive | Negative | - | - | - | - | - |
| 86 | *Puntius titeya* | Positive | Positive | *F. covae* | - | + | - | - |
| 87 | *Puntius titeya* | Positive | Positive | *F. covae* | - | + | - | - |
| 88 | *Puntius titeya* | Positive | Positive | *F. covae* | - | + | - | - |
| 89 | *Lepisosteus oculatus* | Positive | Positive | *F. covae* | - | + | - | - |
| 90 | *Eigenmannia virescens* | Positive | Positive | *F. covae* | - | + | - | - |
| 93 | *Stigmatogobius sadanundio* | Positive | Positive | *F. covae* | - | + | - | - |
| 94 | *Stigmatogobius sadanundio* | Positive | Positive | *F. covae* | - | + | - | - |
| 96 | *Trichogaster microlepis* | Positive | Positive | *F. covae* | - | + | - | - |
| 105 | *Melanochromis auratus* | Positive | Negative | - | - | - | - | - |
| 115 | *Hyphessobrycon ecques* | Positive | Negative | - | - | - | - | - |
| 116 | *Ancistrus cirrhosus* | Positive | Positive | *F. davisii* | - | - | + | - |
| 120 | *Hyphessobrycon herbertaxelrodi* | Positive | Negative | - | - | - | - | - |
| 121 | *Cichla pinima* | Positive | Negative | - | - | - | - | - |
|  |  | 61 | 32 (52,5%) Negative | 29 (47.5%)  Positive | 0 | 27 (44.3%) | 2 (3.3%) | 0 |
